# Cases of Tetanus Successfully Treated†La Clinica Veterinaria.

**Published:** 1885-01

**Authors:** 


### III.—CASES OF TETANUS SUCCESSFULLY TREATED.†

M. Stazzi, V.S., of Brescia, reports that of ten cases of tetanus which he has treated among horses, mules, and asses, seven were successful. His treatment is as follows: the application of an enormous blister to the spinal column, extending from the neck to the tail; hypodermic injections daily of morphine, varying from 30 to 80 centigrammes according to the force of the malady; and enemas containing 15 grammes of extract of belladonna.

The hypodermic injections of morphine until complete cessation of tetanic convulsions is attained, which is generally about the second or third day of the disease. From this time onward 5 grammes of extract of belladonna should be administered. The stable should be kept dark and quiet.

Paralysis of the intestines sometimes occurs during the administration of the medicines, and in this event they should be suspended for a few days, to be administered again when the paralysis ceases.

---

† La Clinica Veterinaria.

M. Trevisi, V.S., of Asolo, has had equal success under a different plan of treatment. Besides the injections of morphine, he uses inhalations of ether and chloroform, and frictions of the ointment of cyanide of potassium on the jaws and along the spine. He also recommends absolute tranquillity and darkness of the stable.

M. Cracos, V.S., of Lucera, uses hypodermic injections of morphine over each jaw, with inhalations of ether and chloroform, enemas, and a dark quiet stable, and generally brings about a cure on the fourth day.

